# Supplementary material for: Pneumococcal Vaccination Coverage and Uptake Among Adults in Switzerland: A Nationwide Cross-Sectional Study of Vaccination Records
Source: Front Public Health. 2022 Jan 31;9:759602. doi: 10.3389/fpubh.2021.759602 (PMC8841552; doi:10.3389/fpubh.2021.759602)
Supplement: Supplementary file 1 [file Table_1.docx]

**Supplementary Table 1.** Swiss Large Regions and Integrated Cantons

| **Large Region** | **Integrated Cantons** |
| --- | --- |
| Lake Geneva | Geneva, Vaud, Valais |
| Ticino | Ticino |
| Midland Switzerland | Bern, Freiburg, Jura, Solothurn, Neuchâtel |
| Northwest Switzerland | Aargau, Basel-Landschaft, Basel-Stadt |
| Central Switzerland | Lucerne, Nidwalden, Obwalden, Schwyz, Uri, Zug |
| East Switzerland | Appenzell-Ausserrhoden, Appenzell-Innerrhoden, Glarus, Graubunden, Schaffhausen, St. Gallen, Thurgau |
| Zurich | Zurich |
