# Supplementary material for: Pneumococcal Vaccination Coverage and Uptake Among Adults in Switzerland: A Nationwide Cross-Sectional Study of Vaccination Records
Source: Front Public Health. 2022 Jan 31;9:759602. doi: 10.3389/fpubh.2021.759602 (PMC8841552; doi:10.3389/fpubh.2021.759602)
Supplement: Supplementary file 2 [file Table_2.docx]

**Supplementary Table 2.** Swiss Linguistic Regions by Canton

| **Linguistic Region** | **Cantons** |
| --- | --- |
| German | Aargau, Appenzell Ausserrhoden, Appenzell Innerrhoden, Basel-Landschaft, Basel-Stadt, Bern*, Freiburg*, Glarus, Graubunden**, Jura*, Lucerne, Nidwalden, Obwalden, Schaffhausen, Solothurn, St. Gallen, Thurgau, Uri, Valais*, Schwyz, Zug, Zurich |
| French | Bern*, Freiburg*, Geneva, Jura*, Neuchâtel, Valais*, Vaud |
| Italian | Graubunden**, Ticino |

*Canton has both official German and French-speaking communities

**Canton has both official German and Italian-speaking communities
