# Supplementary material for: Pneumococcal Vaccination Coverage and Uptake Among Adults in Switzerland: A Nationwide Cross-Sectional Study of Vaccination Records
Source: Front Public Health. 2022 Jan 31;9:759602. doi: 10.3389/fpubh.2021.759602 (PMC8841552; doi:10.3389/fpubh.2021.759602)
Supplement: Supplementary file 3 [file Table_3.docx]

**Supplementary Table 3.** Distribution of Self-Reported Risk Factors by Gender and Age

| **Disease** | **% of Respondents**  **(95% CI)** | **%Male**  **(95% CI)** | **%Female**  **(95% CI)** | **%18-39**  **(95% CI)** | **%40-64**  **(95% CI)** | **%65-85**  **(95% CI)** |
| --- | --- | --- | --- | --- | --- | --- |
| No Risk Reported | 83.76  (82.51-85.01) | 82.49  (80.55-84.44) | 85.02  (83.43-86.59) | 89.83  (87.78-91.88) | 84.64  (82.60-86.68) | 71.38  (69.04-73.73) |
| Asthma | 7.92  (6.96-8.88) | 7.37  (5.93-8.81) | 8.47  (7.20-9.74) | 7.35  (5.57-9.14) | 7.40  (5.95-8.85) | 10.02  (8.41-11.63) |
| Immunosupression | 2.22  (1.74-2.71) | 1.47  (0.87-2.08) | 2.97  (2.21-3.72) | 1.02  (0.39-1.65) | 2.68  (1.81-3.55) | 3.33  (2.37-4.29) |
| Diabetes | 2.88  (2.36-3.40) | 3.79  (2.91-4.66) | 1.99  (1.43-2.56) | 0.76  (0.22-1.29) | 2.56  (1.64-3.48) | 7.26  (5.96-8.56) |
| Heart/Liver/Kidney Disease | 5.34  (4.62-6.06) | 6.83  (5.64-8.03) | 3.87  (3.07-4.66) | 1.44  (0.61-2.27) | 4.93  (3.68-6.18) | 12.97  (11.24-14.70) |
| >1 Health Risk | 1.67  (1.26-2.07) | 1.55  (0.98-2.12) | 1.78  (1.22-2.34) | 0.40  (-0.03-0.80) | 1.63  (0.93-2.33) | 3.94  (2.88-4.99) |
