# Supplementary figures and images for: Pneumococcal Vaccination Coverage and Uptake Among Adults in Switzerland: A Nationwide Cross-Sectional Study of Vaccination Records
Source: Front Public Health. 2022 Jan 31;9:759602. doi: 10.3389/fpubh.2021.759602 (PMC8841552; doi:10.3389/fpubh.2021.759602)

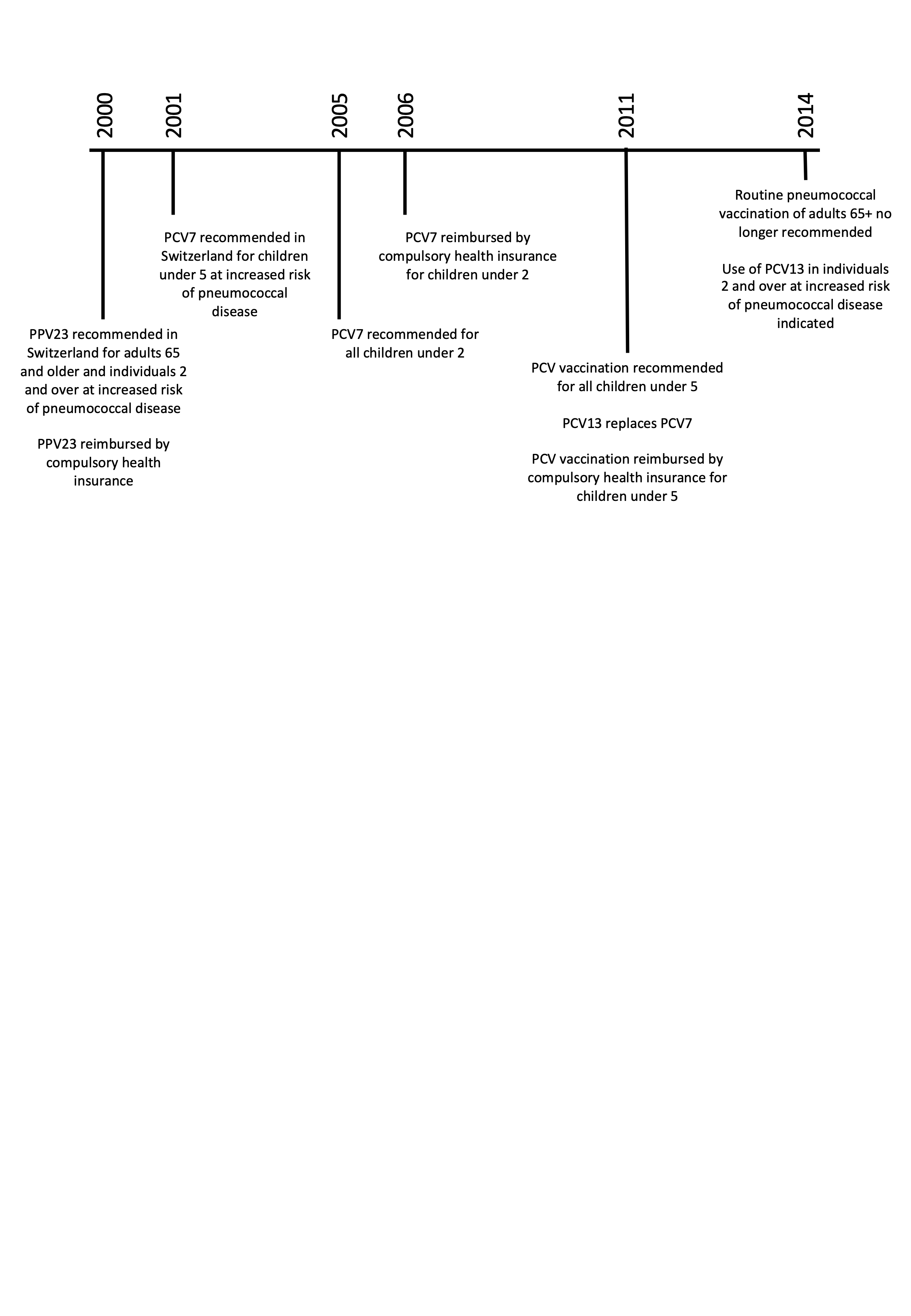

Supplement: Supplementary file 4 [file Image_1.TIFF]

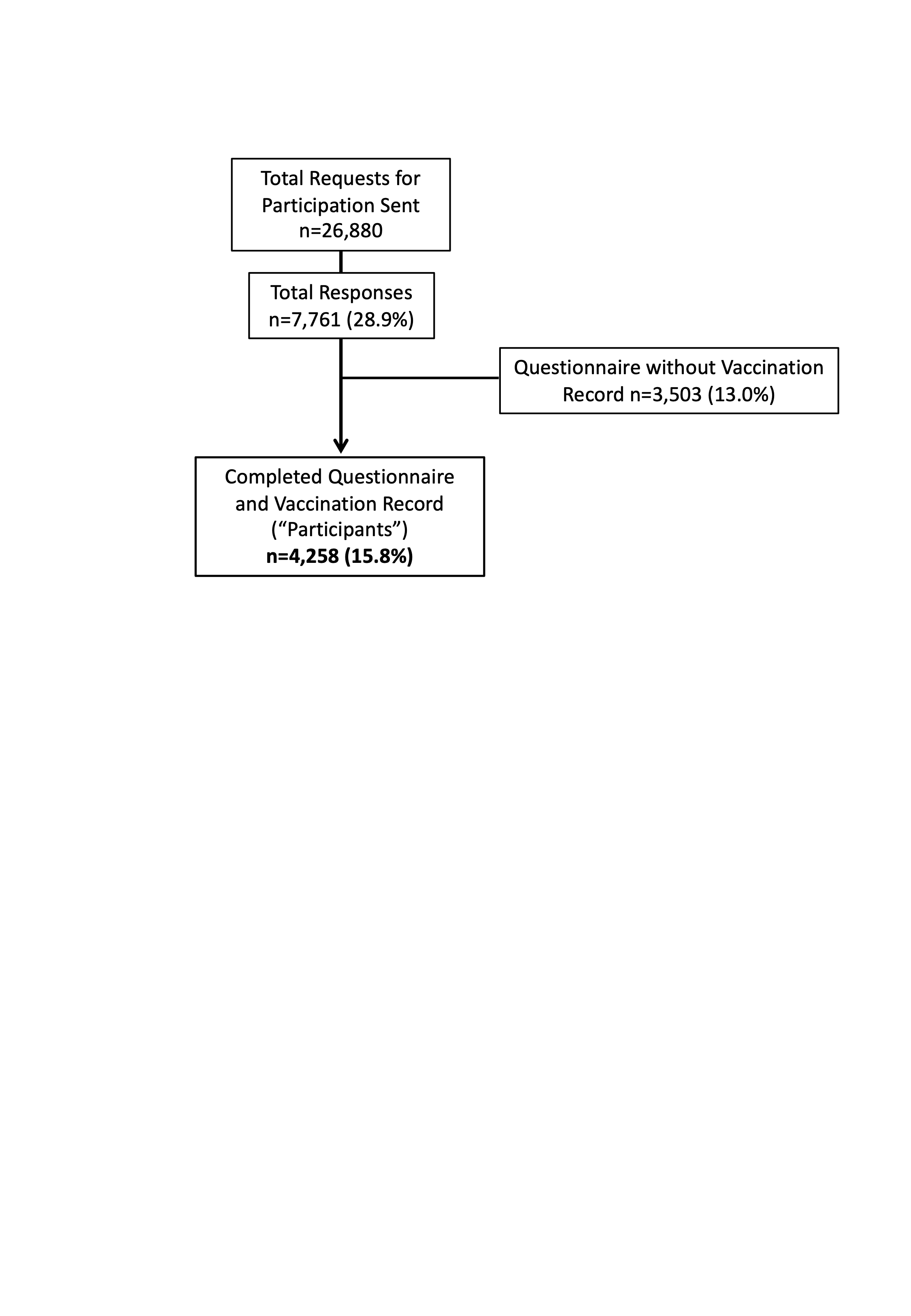

Supplement: Supplementary file 5 [file Image_2.TIFF]

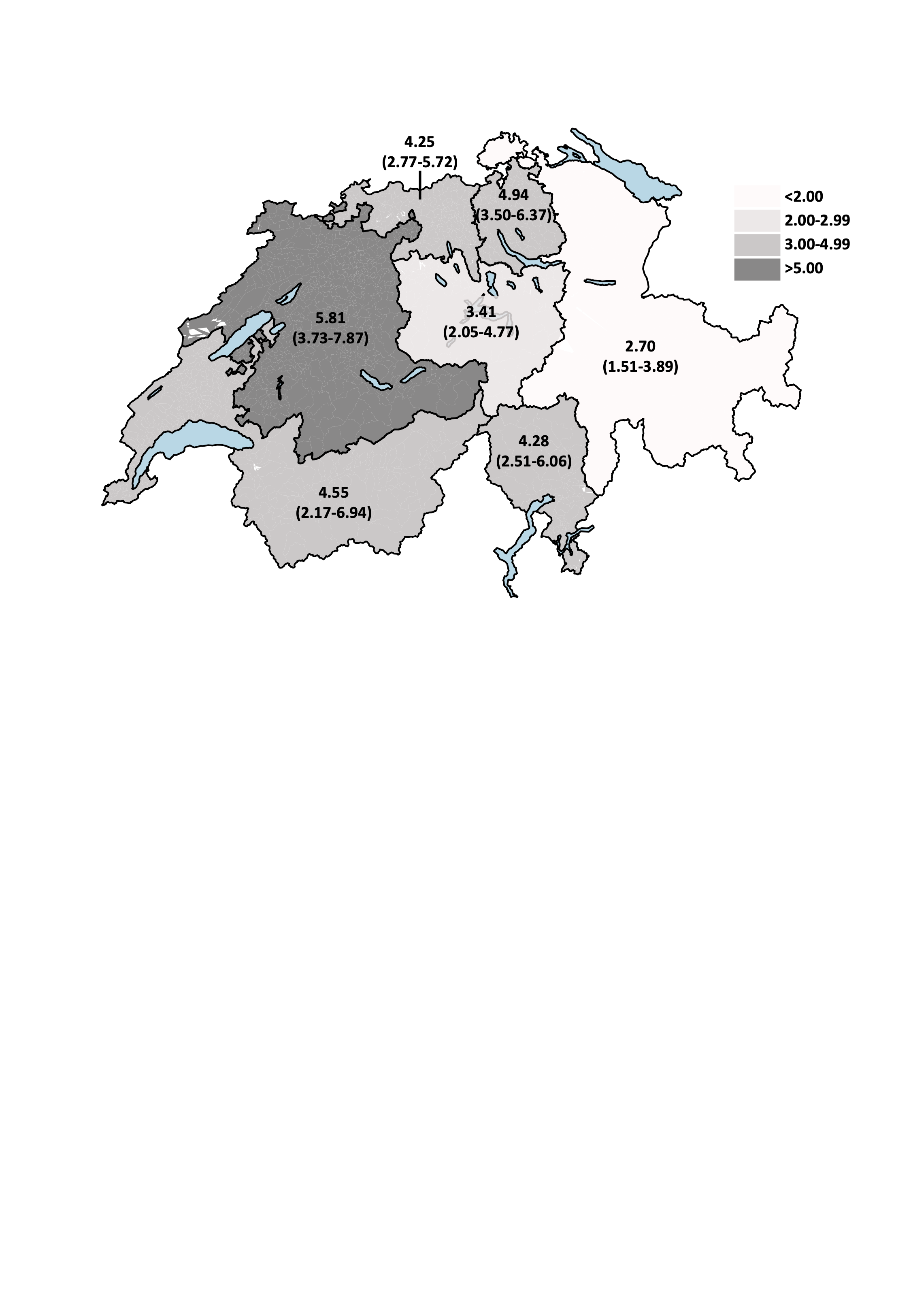

Supplement: Supplementary file 6 [file Image_3.TIFF]
