# Supplementary material for: Pneumococcal Vaccination Coverage and Uptake Among Adults in Switzerland: A Nationwide Cross-Sectional Study of Vaccination Records
Source: Front Public Health. 2022 Jan 31;9:759602. doi: 10.3389/fpubh.2021.759602 (PMC8841552; doi:10.3389/fpubh.2021.759602)
Supplement: Supplementary file 7 [file Data_Sheet_1.docx]

Supplementary Figure 1. Timeline of Pneumococcal Vaccination Recommendations in Switzerland.

**Supplementary Figure 2.** Flow Chart of Study Respondents. A total of 26,880 requests for participation were sent to randomly selected individuals by mail. Individuals were included in the study if they submitted both a completed questionnaire and a copy of their vaccination record

**Supplementary Figure 3.** Nationwide Adult Pneumococcal Vaccination Coverage by Large Region. The fraction of adults 18-85 vaccinated for pneumococcus in each Swiss large region. (n=4258, p=0.11, Linear regression followed by Adjusted Wald test with Bonferroni’s adjustment).
